# Monkeys Are More Patient in a Foraging Task than in a Standard Intertemporal Choice Task

**DOI:** 10.1371/journal.pone.0117057

**Published:** 2015-02-11

**Authors:** Tommy C. Blanchard, Benjamin Y. Hayden

**Affiliations:** Department of Brain and Cognitive Sciences and Center for Visual Science, University of Rochester, Rochester, New York, United States of America; Duke University, UNITED STATES

## Abstract

Studies of animal impulsivity generally find steep subjective devaluation, or discounting, of delayed rewards – often on the order of a 50% reduction in value in a few seconds. Because such steep discounting is highly disfavored in evolutionary models of time preference, we hypothesize that discounting tasks provide a poor measure of animals’ true time preferences. One prediction of this hypothesis is that estimates of time preferences based on these tasks will lack *external validity*, i.e. fail to predict time preferences in other contexts. We examined choices made by four rhesus monkeys in a computerized patch-leaving foraging task interleaved with a standard intertemporal choice task. Monkeys were significantly more patient in the foraging task than in the intertemporal choice task. Patch-leaving behavior was well fit by parameter-free optimal foraging equations but poorly fit by the hyperbolic discount parameter obtained from the intertemporal choice task. Day-to-day variation in time preferences across the two tasks was uncorrelated with each other. These data are consistent with the conjecture that seemingly impulsive behavior in animals is an artifact of their difficulty understanding the structure of intertemporal choice tasks, and support the idea that animals are more efficient rate maximizers in the multi-second range than intertemporal choice tasks would suggest.

## Introduction

While it is often thought of as a human problem, impatience is frequently studied in animals, both as a model of human impulsivity, and to understand animals’ own psychology [1,2]. On each trial of the widely used *intertemporal choice task*, animals choose between a shorter sooner (SS) option and a larger later (LL) one [3,4]. Choice of the SS is taken to be a sign of impatience; aggregate choice behavior provides a discount factor that quantifies the rate at which subjective value declines as a function of its delay.

Data from the intertemporal choice task generally show animals discount rewards by 50% within a few seconds [5–8] (although some apes have been shown to discount rewards on the order of minutes, e.g. [9]). However, this portrait of impulsive animals is at odds with basic evolutionary accounts, which predict animals should be efficient rate maximizers over short spans of time [10–12]. One possible explanation for this inconsistency is that the intertemporal choice task provides either a biased or inaccurate measure of time preferences [6,7,12]. If this is the case, then discount factors obtained from this task may have poor *external validity*.

**Figure 1:**
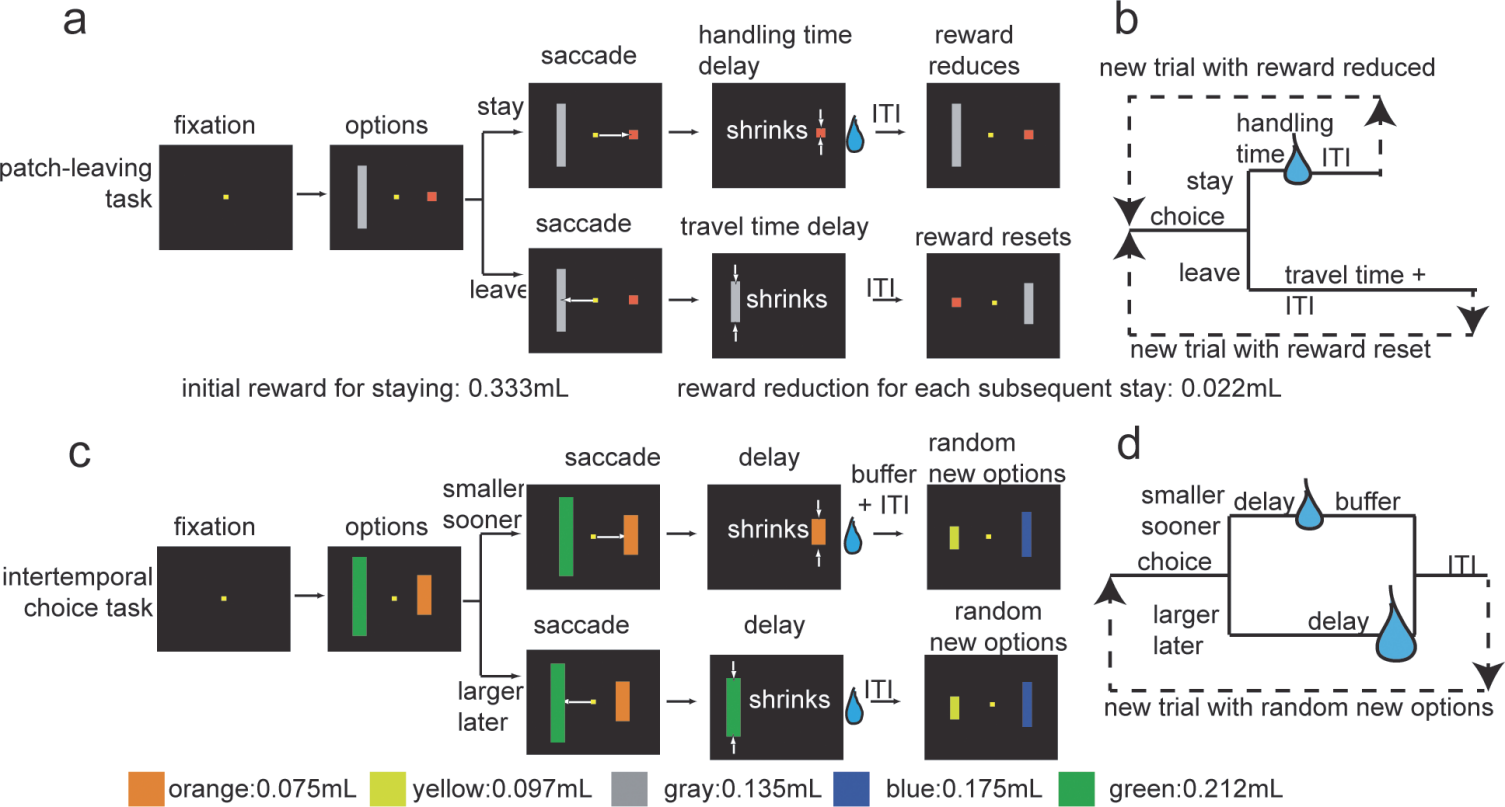
Task diagrams. Structure of (a-b) patch-leaving and (c-d) intertemporal choice tasks. Following fixation, two options appeared and subject chose one via saccade. Bar height indicated delay. In the patch-leaving task, color differentiated leave and stay options; in the discounting task, color identified reward associated with the choice.

To test the external validity of discount factors measured in intertemporal choice tasks, we trained monkeys to perform both the intertemporal choice task and a standard foraging problem, the *patch-leaving task*, in interleaved blocks within single sessions (**Fig. 1**). Like the intertemporal choice task, the patch-leaving task involves trading off delays and reward values [13]. On each trial, the subject chooses between staying in or leaving a patch; staying results in a short delay and a reward, but reduces the reward for subsequent staying choices. Leaving a patch results in a longer delay and no reward, but resets the reward to its maximum amount. Foraging theory shows that the optimal (rate-maximizing) solution to this problem is to leave the patch at the point that where the marginal intake from staying falls to match that obtained from leaving [10]. Such behavior should be observed if animals have neutral time preferences; however, if animals discount delayed rewards, they ought to stay in the patch longer than foraging theory dictates, since the rewards associated with leaving are greatly delayed relative to the rewards associated with staying. We therefore assessed the external validity of the intertemporal choice task by testing how well measured discount factors predicted choices in the patch-leaving task.

## Materials and Methods

### General methods

All animal procedures were approved by the University of Rochester Animal Care and Use Committee and were conducted in compliance with the Public Health Service’s Guide for the Care and Use of Animals.

Four male rhesus macaques (*Macaca mulatta*), aged 6–8 years and weighting 6–8kg served as subjects. All subjects had previous experience in similar decision-making tasks. All experiments were run in the lab between the hours of 9am-5pm. Subjects were pair-housed, with each pair having access to two 470 cm^2^ cages, and one larger 610 cm^2^ pen. Subjects had access to a minimum of two enrichment items while in their home cages (examples of enrichment items include tire swings, mirrors, and stuffed animals). Subjects had full access to food (LabDiet 5045, ad libitum) while in their home cages. Subjects were water restricted, and received at minimum 20mL per kg of water per day, although in practice they received close to double this amount in the lab as a result of our experiments. No subjects were sacrificed as a result of these experiments. No animals were physically harmed in the course of these experiments. Animals were weighed twice per week to monitor weight fluctuations due to fluid restrictions.

Visual stimuli were colored rectangles on a computer monitor (see **Fig. 1**). Stimuli were controlled by Matlab with Psychtoolbox and Eyelink Toolbox. A solenoid valve controlled the delivery duration of fluid rewards. Eye positions were sampled by a camera system (SR Research, Osgoode, ON, Canada). A small mount was used to facilitate maintenance of head position during performance. These were surgically implanted for previous experiments and while the animals were under anesthetic.

Three of our monkeys (subjects C, J & H) had previous extensive training on the intertemporal choice task from a previous study shortly before we began data collection for the present study [6]. This training provides additional assurance that the monkeys had time to learn the task parameters in the intertemporal choice task (cf. 6). We ensured that all three monkeys showed sensitivity to delay length and reward size when put back on the intertemporal choice task, and that their behavior was stable prior to data collection for this study. The fourth monkey (subject B) did not have prior experience with the intertemporal choice task. He was first trained on the intertemporal choice task for 8 days, during which he completed 10,067 trials. All four monkeys were trained on the patch-leaving task, which they learned rapidly (#days/#blocks/#trials for subject B: 7/5077/883; subject C: 5/3395/853; subject H: 4/5786/543; subject J: 5/3173/271). This rapid learning of the patch-leaving task confirms our earlier demonstration that monkeys can learn patch-leaving tasks within a day or two [13]. Following this training on each task separately, subjects performed three additional days of training with the tasks interleaved in the same manner they were during data collection. All animals exhibited stable behavior (i.e. similar sensitivity to reward and delays between days) before data collection began. During data collection, animals performed 500 trials of the patch leaving task (approximately 50–70 blocks, depending on the condition), then 200 trials of the intertemporal choice task, then the patch leaving task again until they had received their full allotment of fluid for the day (usually another 500 trials).

### Intertemporal choice task

Each trial began with a 100 msec fixation period followed by the appearance of colored rectangles (80 pixels wide) located 275 pixels to the left and right of the central spot. Rectangle color indicated its offered reward size (orange = 75, yellow = 97, grey = 135, blue = 175, and green = 212 microliters). Rectangle height indicated its delay (1 to 300 pixels tall, 0 to 6 seconds). Monkeys selected options by a saccade. During the delay that was chosen, the bar shrunk (50 pixels/sec), providing a visible reminder of the progress through the delay. During the delay period, gaze was unconstrained. The reward occurred once the rectangle shrunk entirely. The duration of the adjusting post-reward buffer was chosen so that total trial length was 6 seconds, regardless of choice. The screen was blank throughout the post-reward buffer and the inter-trial interval. The inter-trial interval was 1 second. On each trial, rewards were chosen randomly such that the two rewards were not equivalent. Delays were chosen randomly from a uniform distribution, and the shorter of the delays was assigned to the option with the lower reward, so that choices were always between a shorter-sooner and a larger-later option.

### Patch leaving task

We used a computerized version of the patch-leaving problem from foraging theory that was developed for an earlier study [13]. Each trial began with a 100 msec fixation period followed by the appearance of colored rectangles (80 pixels wide) located 275 pixels to the left and right of the central spot.

The *stay in patch* option was a short red rectangle and the *leave patch* option was a longer grey rectangle. Bar height indicated the length of the delay associated with choosing that option. On the first trial in each block, the stay in patch option yielded a short delay (0.6 seconds) and a large reward (333 microliters). Each subsequent choice of the stay option yielded the same delay and a smaller reward. Decrease in solenoid open time was linear; true volume yielded was close to linear (approximately 22 microliters). We measured true reward amounts from each solenoid open time, and used these values in our calculations. Choosing the leave patch option resulted in no reward, a longer delay and an end to the block. The leave option delay was chosen at random at the beginning of a session and remained constant throughout the session, and was either 4.8, 8, or 12.8 seconds. The inter-trial interval was 1 second. Because the optimal behavior in this task is sensitive to all delays, we used in our foraging equations the median total time for each trial type for use in our calculations.

### Discounting factor calculations

Experimenters typically use a hyperbolic discounting equation to model temporal preferences [5–8]. This equation, describing a hyperbolic curve, is fit to each subject’s data and can be used to predict subjects’ preferences for delayed rewards. Discount factors used here came from the hyperbolic discounting equation:

$$v_{discounted}=\frac{v_{actual}}{1+\text{​}k*\text{​}D}$$

Where *v*_*discounted*_ is the subjective, discounted value, *v*_*actual*_ is the actual reward amount, *D* is the delay to the reward, and *k* is the fitted discount factor. Discount factors were computed using standard methods [6,14].

### Value index calculations

To compare the theoretical predictions of discounting theory and optimal foraging theory to our subjects’ behavior, we computed value indices for each theory. These are scalar quantities that represent indices indicate the predicted normalized subjective value of staying versus leaving. A positive value index indicates that the model predicts that staying is more valuable than leaving; a negative value index indicates that the model predicts leaving is more valuable than staying. Thus, when calculated for the trials on which the animal chose to leave, these indices serve as a measurement of how much the theories’ predicted preferences differ from the actual subject’s behavior.

To determine subjective value of the stay and leave options in the patch-leaving task according to discounting theory, we used the *k* parameter derived from the intertemporal choice task for each day. We then calculated the value of staying and leaving according to the hyperbolic discounting formula. Because the length of stay and leave trials were not equivalent, we added the discounted value of future trials. Since this is an infinite sum, we only added the values of future trials out until the value of the future trial was less than 0.1% of the difference between staying and leaving. The *discounting value index* reported was (on the trial the animal chose to leave the patch):

$$v_{index}=\frac{v_{stay}-v_{leave}}{v_{stay}+v_{leave}}$$

The value index according to the optimal foraging theory was the difference between the reward rate (reward obtained divided by the total time taken to acquire that reward, including the time taken to leave the patch) obtained in the block, and the maximum reward rate possible. The *foraging value index* reported was:

$$v_{index}=\frac{v_{obtained}-v_{optimal}}{v_{optimal}+v_{obtained}}$$

To match the discounting value index, which is positive when the animal leaves the patch earlier than predicted and negative when the animal leaves the patch later than predicted, we took the absolute value of this index when the animal left the patch too early.

## Results

### Performance in the intertemporal choice task

We collected a large set of intertemporal choice data in four monkeys (#sessions/#trials for ***subject B***: 30/5159, ***subject C***: 12/2463, ***subject H***: 19/3386, ***subject J***: 17/3782). Each trial pitted an option with a larger, but more delayed reward (larger-later option, LL) against an option with a smaller, but less delayed reward (smaller-sooner option, SS). The reward sizes and delays for each option were chosen randomly each trial (see Methods).

**Figure 2:**
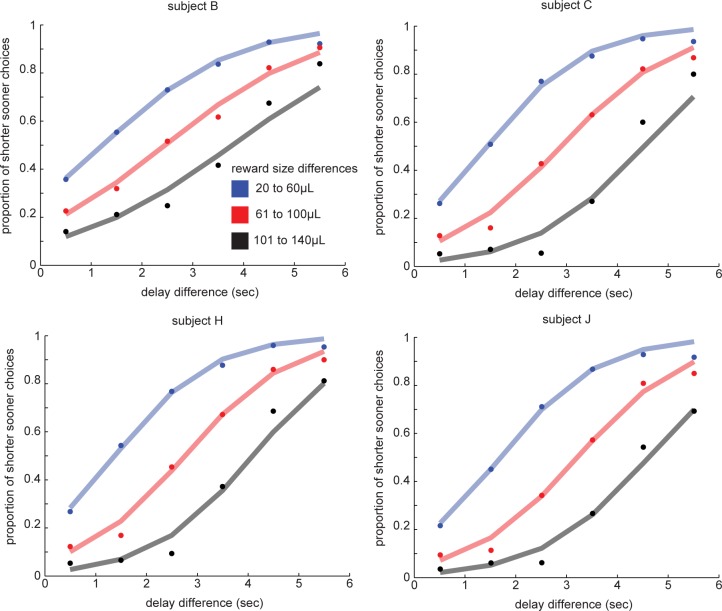
Discounting task behavior. Animals were more likely to choose the Smaller-Sooner option when the reward size difference between the options was small or the delay difference was large. Points indicate binned data, lines are best fit logistic regression lines.

If animals understand the task, we would expect their choices to depend on the delay and reward size differences between the two options. To confirm that this is the case, we performed a logistic regression on the choice data for each subject, which showed that our subjects’ choices indeed did depend on both delay and reward size (*P*<0.0001 for each variable for each subject, logistic regression of choice of SS onto reward size and delay differences between the options; **Fig. 2**).

Behavior on intertemporal choice tasks is typically fit with a hyperbolic discounting curve [5–8]. This curve fit provides a discount factor, k, that summarizes how steeply the animal discounts delayed rewards, and thus can be used to predict the subjective value a delayed reward has for the animal. A higher k-value indicates a steeper discounting curve, which is typically interpreted as indicating higher impulsivity. We computed discount factors for all four subjects (hyperbolic k-values in units of seconds^−1^ for ***subject B***: 0.768, ***subject C***: 0.426, ***subject H***: 0.496, ***subject J***: 0.380). These values are within the range found in our own studies and in previous studies of discounting in rhesus monkeys [5–8].

### Performance in the patch-leaving foraging task

**Figure 3:**
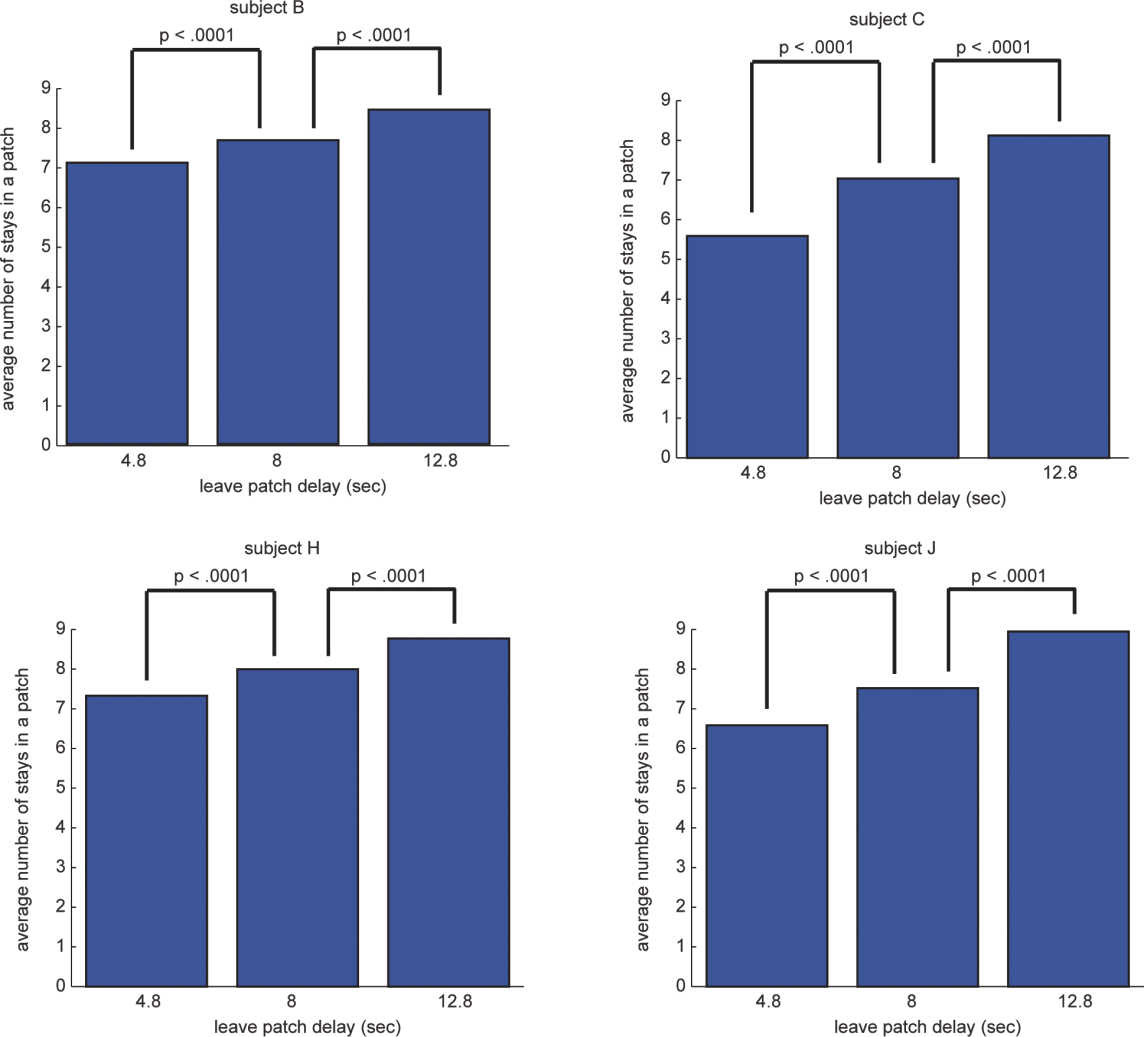
Patch leaving behavior. Animals chose to stay longer in the patch when the delay incurred by leaving the patch was longer. Bars indicate mean stays in a patch for each condition, P-values are for t-tests comparing number of stays for each condition.

We collected patch-leaving data in matched interleaved sessions with intertemporal choice data (#sessions/#blocks for ***subject B***: 30/3366, ***subject C***: 12/1507, ***subject H***: 19/2377, ***subject J***: 17/2185). Foraging theory shows that animals should be sensitive to the delay associated with leaving a patch – the longer this delay, the longer animals should stay in the patch. Consistent with this prediction, all four of our animals tended to stay longer in the patch during blocks with longer search times (*P*<0.0001 in all cases, **Fig. 3**).

**Figure 4:**
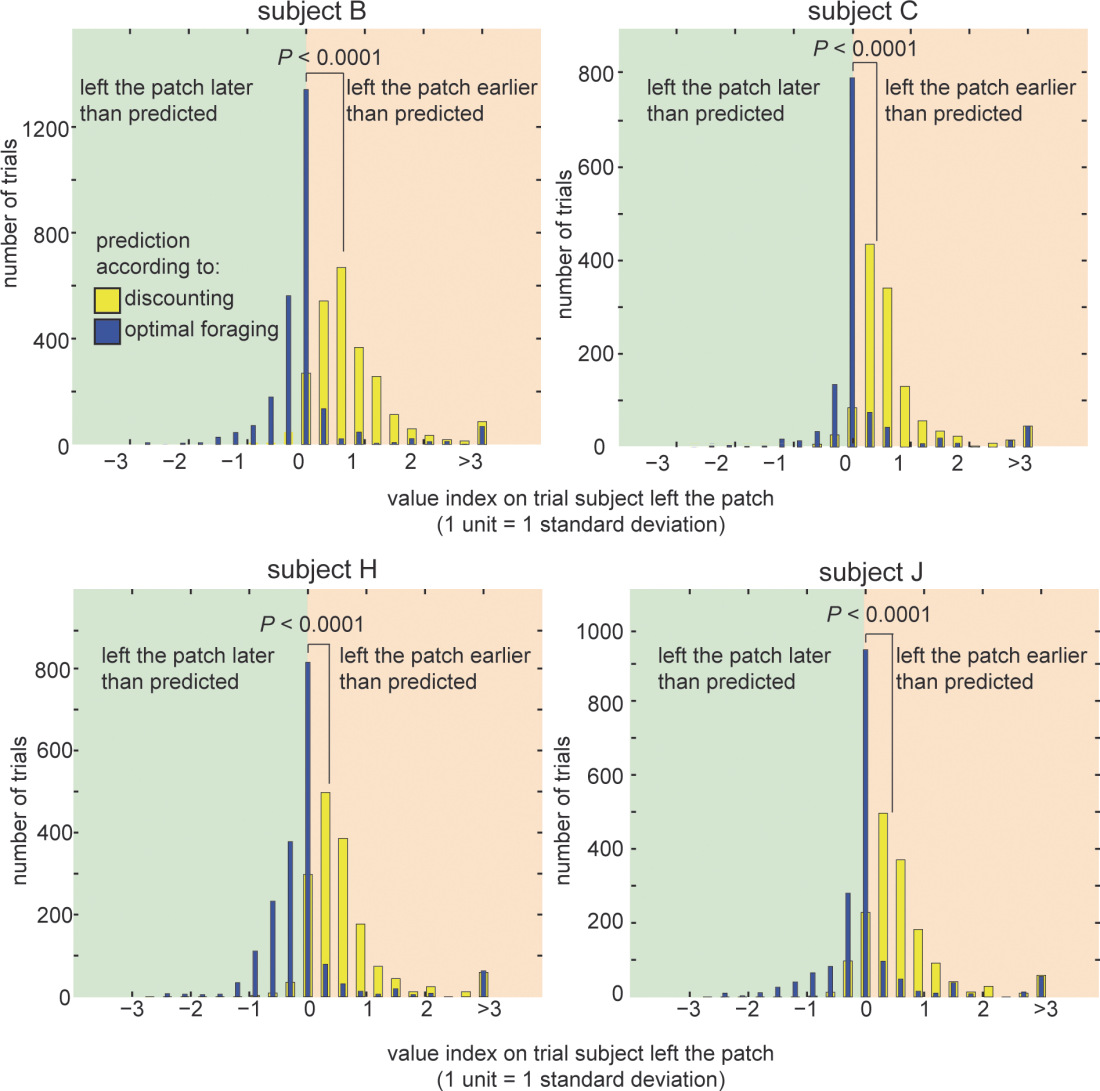
Animals are more patient in the patch-leaving task than their intertemporal choice task behavior predicts. Histogram showing the distribution of (blue) foraging value indices and (yellow) discounting value indices on trials where the animal chose to leave. Positive values indicate leaving was earlier than predicted by the theory; negative numbers indicate leaving was later than predicted. The mean of the yellow histogram being significantly positive indicates that animals tended to leave earlier than predicted by discounted value models, (i.e. they showed a greater willingness to wait for a larger reward than expected). The mean of the blue histogram being significantly closer to 0 indicates that the optimal foraging theory models predicted behavior more accurately than discounted value models. P-values indicate the results of a 2-sample t-test performed on the two distributions.

We computed monkeys’ performance in the patch-leaving task relative to prescriptions of foraging theory [10]. To do so, we calculated a *foraging value index* that serves as a measurement of how much the animals’ behavior differs from the prescriptions of optimal foraging theory (see **Methods**). Briefly, a negative foraging value index indicates the animal is leaving the patch earlier than prescribed by optimal foraging theory. A positive index indicates staying in the patch longer than prescribed by optimal foraging theory, and may be attributed to discounting. A foraging value index of 0 indicates optimal performance. For one of our subjects, the foraging value index was not statistically different from zero, indicating there was no statistical difference between behavior and the prescriptions of optimal foraging theory (***subject B***: mean foraging value index MFVI = 0.014, *P* = 0.425, Student’s *t*-test; value difference units are given in standard deviations). Two showed a weak but significant bias towards early leaving (***subject J***, MFVI = 0.0422, *P* = 0.049; ***subject C***, MFVI = 0.168, *P*<0.0001). The final subject showed a weak but still significant tendency to stay in the patch too long (***subject H***: MFVI = −0.044, *P* = 0.044, **Fig. 4**).

We noticed the distribution of value differences was skewed by extreme positive values, most likely caused by the animal (perhaps unintentionally) leaving the patch on the first trial of the block. Thus, we ran the same analysis as above, excluding values three standard deviations from the median (blocks excluded, n = 82, 52, 74, 63 for Subject B, C, H & J, respectively). With those blocks excluded, three of our animals showed a tendency to stay in the patch too long (subject B, MFVI = −0.116, *P*<0.0001; Subject H, MFVI = −0.189, *P*<0.0001; Subject J, MFVI = −0.093, *P*<0.0001), and the final subject was not significantly different from 0 (Subject C, MFVI = 0.010, *P* = 0.464). We excluded the outliers from the remainder of our analyses, although their inclusion does not qualitatively change any of our claims here.

### Discount factors lack external validity

If an animal’s discount factor measures true time preferences, the animal should choose foraging options according to their discounted values. We therefore created a *discounting value index* that serves as a measure of how much the animals’ behavior differs from the predictions of discounting theory. We calculated this index using the animals’ measured discount rates in the intertemporal choice task to predict the value they would assign to staying or leaving the patch. Our discounting value index was the predicted subjective value of staying minus the predicted subjective value of leaving (see **Methods**). A discounting decision-maker should thus choose to leave a patch when the discounted value of staying drops below the discounted value of leaving. By calculating the discounting value index on trials that the animal actually chose to leave, we can compare this prediction to the animals’ actual behavior. A positive discounting value index on these trials indicates that the animal left earlier than predicted by discounting theory, and a negative value indicates that the animal left later than predicted. Because leaving leads to a longer delay to the next reward, a positive discounting value index indicates that the subject was more patient than predicted by their behavior in the intertemporal choice task. All four subjects left patches much earlier than predicted by this *discounted foraging model* (***subject B***: Mean Discounting Value Index, MDVI = 0.815; ***subject C***: MDVI = 1.069; ***subject H***: MDVI = 0.668; ***subject J***: MDVI = 0.704; all are greater than zero, *P*<0.0001, Student’s *t*-test; value difference units are given in standard deviations; **Fig. 4**).

Because these values are standardized (units are in standard deviations), they are directly comparable to those based on optimal foraging predictions above. For all four animals, optimal foraging was significantly better predictor of behavior than the discount factor derived from the intertemporal choice task (i.e. value difference was significantly closer to 0; *P*<0.0001 in all cases, 2-sample *t*-test on the value differences for the two models but with the signs flipped for animals who had a negative MVD for foraging theory). It is worth emphasizing that the foraging model makes more accurate predictions despite the fact that it involves no free parameters, but is derived from first principles [10].

### No correlation between day-to-day variations in performance in the two tasks

Do day-to-day fluctuations in intertemporal choice task performance predict day-to-day fluctuations in patch-leaving times? To answer this question, we calculated the correlation between the discount factor for each day and the leaving time (specifically, the difference between the optimal and observed leaving times). If the discount factor measures an intrinsic time preference, then large discount factors from the intertemporal choice task should predict over-staying in the patch-leaving task, and we would see a correlation between these variables. We found no correlation in any of our subjects, or even a consistent trend in the sign of the correlation across subjects (***subject B***: r = −0.219, *P* = 0.106; ***subject C***: r = 0.233, *P* = 0.273; ***subject H***: r = −0.299, *P* = 0.0814; ***subject J*:** r = 0.236, *P* = 0.180;). Thus, despite the similarities of the tasks, variations in time preference observed in the intertemporal choice do not predict variations in time preference observed in the patch-leaving task.

## Discussion

We compared time preferences of four rhesus monkeys in an intertemporal choice task and a patch-leaving foraging task. Monkeys showed steep discounting in the intertemporal choice task but much more patient behavior in the foraging task. We found that day-to-day variation in time preferences as measured by intertemporal choice task performance had no relationship with variation in time preferences as measured by foraging task performance. We conclude that discount factors derived from the intertemporal choice task have poor external validity to even superficially similar foraging tasks. Admittedly, it is common to recalibrate discount factors across tasks and conditions. However, we feel that this practice raises troubling questions about the meaning of discount factors: if they measure some intrinsic time preference, then this time preference ought to be stable across tasks. These questions are particularly salient given the recent doubts that have been raised concerning animals abilities to understand the design of intertemporal choice tasks, even with extensive training [6,7,15,16].

If time preferences measured by foraging tasks and intertemporal choice tasks are unrelated, which task measures true time preferences? We believe that the present results, combined with previous findings, suggest that foraging tasks provide a more accurate measure of an animals’ true time preferences. We have three main reasons: 1) the foraging task used is thought to be mathematically and intuitively closer to the types of foraging problems monkeys evolved to solve [10]; 2) our results here show that behavior in the foraging task come very close to the a priori predictions of foraging theory [10]; and 3) previous evidence suggests that monkeys have a poor understanding of the temporal structure of intertemporal choice tasks [6,7,15,16].

Previous work indicates that, even within the context of the intertemporal choice task, contextual factors affect measured discount rates [17–19]. For example, a reward being visually present motivates choices of the larger-later reward due to animals’ propensity to reach for larger rewards [18,19]. These issues have led some to propose alternative, more intuitive tasks to measure delay of gratification in non-human primates [20]. Our work here shows that even a minor difference in task structure can lead to not only different temporal preferences, but temporal preferences that bear no obvious relationship to behavior in the standard intertemporal choice task. Due to the similar temporal structure between the two tasks, the lack of a relationship between temporal preferences between the two tasks shows that the temporal preferences measured in the intertemporal choice task are extremely limited in the contexts they apply to.

Few studies have tested the external validity of the intertemporal choice parameters in animals. One previous study showed that behavior in the intertemporal choice task is only weakly correlated with behavior on another measure of self-control, the accumulation task, in which food items accumulate in front of the subject at a fixed rate and the accumulation stops when the subject reaches for the food items [21]. Two other studies report dissimilar performance in foraging and intertemporal choice tasks, a bias that cannot be explained by a pure short-term rate-maximizing heuristic [22,23]. More recently, we have found that monkeys have near-optimal performance in both patch-leaving and diet selection foraging contexts, findings that are inconsistent with monkeys’ known steep discounting [13,24]. Our work here confirms that such disparate measures of time preferences can be observed in the same subjects within the same sessions, and suggest that the two tasks do not call upon a single domain-general time preference function.

Why does the intertemporal choice task have such poor external validity? We and others have previously argued that intertemporal choice tasks pose a special learning problem for animals: they cannot understand the structure of the adjusting post-reward buffer without special cueing [6,7,15,16]. This leads to systematic biasing in the time preference measurements, making animals seem much more impulsive than they actually are [6,7]. Our present work extends these findings, confirming that monkeys are more patient in foraging tasks, and that time preference measurements in intertemporal choice tasks do not have predictive validity even to the similarly structured foraging task.
